# Cardiology knowledge assessment of retrieval-augmented open versus proprietary large language models

**DOI:** 10.1371/journal.pdig.0001029

**Published:** 2026-03-12

**Authors:** Constantine Tarabanis, Shaan Khurshid, Areti Karamanou, Rodo Piperaki, Lucas A. Mavromatis, Aris Hatzimemos, Dimitrios Tachmatzidis, Constantinos Bakogiannis, Vassilios Vassilikos, Patrick T. Ellinor, Lior Jankelson, Evangelos Kalampokis

**Affiliations:** 1 Cardiology Division, Heart and Vascular Institute, Mass General Brigham, Boston, Massachusetts, United States of America; 2 Cardiovascular Disease Initiative, Broad Institute of MIT and Harvard, Cambridge, Massachusetts, United States of America; 3 Telemachus and Irene Demoulas Family Foundation Center for Cardiac Arrhythmias, Massachusetts General Hospital, Boston, Massachusetts, United States of America; 4 Cardiovascular Research Center, Massachusetts General Hospital, Boston, Massachusetts, United States of America; 5 Information Systems Laboratory, University of Macedonia, Thessaloniki, Greece; 6 New York University School of Medicine, New York City, New York, United States of America; 7 3rd Cardiology Department, Hippokrateion University Hospital, Aristotle University of Thessaloniki, Thessaloniki, Greece; 8 Leon H. Charney Division of Cardiology, NYU Langone Health, New York University School of Medicine, New York City, New York, United States of America; Peng Cheng Laboratory, CHINA

## Abstract

To evaluate the performance of open-weight and proprietary LLMs, with and without Retrieval-Augmented Generation (RAG), on cardiology board-style questions and benchmark them against the human average. We tested 14 LLMs (6 open-weight, 8 proprietary) on 449 multiple-choice questions from the American College of Cardiology Self-Assessment Program (ACCSAP). Accuracy was measured as percent correct. RAG was implemented using a knowledge base of 123 guideline and textbook documents. The open-weight model DeepSeek R1 achieved the highest accuracy at 86.9% (95% CI: 83.4–89.7%), outperforming proprietary models and the human average of 78%. GPT 4o (80.9%, 95% CI: 77.0–84.2%) and the commercial platform OpenEvidence (81.3%, 95% CI: 77.4–84.7%) demonstrated similar performance. A positive correlation between model size and performance was observed within model families, but across families, substantial variability persisted among models with similar parameter counts. After RAG, all models improved, and open-weight models like Mistral Large 2 (78.0%, 95% CI: 73.9–81.5) performed comparably to proprietary alternatives like GPT 4o. Large language models (LLMs) are increasingly integrated into clinical workflows, yet their performance in cardiovascular medicine remains insufficiently evaluated. Open-weight models can match or exceed proprietary systems in cardiovascular knowledge, with RAG particularly beneficial for smaller models. Given their transparency, configurability, and potential for local deployment, open-weight models, strategically augmented, represent viable, lower-cost alternatives for clinical applications. Open-weight LLMs demonstrate competency in cardiovascular medicine comparable to or exceeding that of proprietary models, with and without RAG depending on the model.

## Author summary

In this work, we set out to understand how today’s artificial intelligence systems perform when tested on the kind of questions cardiologists face during board examinations. We compared a wide range of large language models, including both “open-weight” models and commercial “proprietary” ones, and also tested whether giving the models access to trusted cardiology textbooks and guidelines could improve their answers. We found that the best open-weight model actually outperformed all of the commercial models we tested, even exceeding the average score of practicing cardiologists. When we gave the models access to medical reference material, nearly all of them improved, with the biggest gains seen in the smaller and weaker models. This shows that careful design and support can allow smaller, more accessible systems to reach high levels of accuracy. Our results suggest that open-weight models, which can be used locally without sending sensitive patient information to outside servers, may be a safe and cost-effective alternative to commercial products. This matters because it could make powerful AI tools more widely available across hospitals and clinics, while also reducing risks related to privacy, transparency, and cost.

## Introduction

Large language models (LLMs) have been proposed to hold transformative potential in cardiovascular medicine [1,2], with applications ranging from automated note generation to clinical consultations [3]. To optimize their performance in medical contexts, various techniques have been developed, including domain-specific fine-tuning and retrieval-based methods [4]. An example of the latter is Retrieval-Augmented Generation (RAG), which aims to improve performance by incorporating relevant content from a provided document set during response generation [4]. However, before these models can be reliably applied in clinical practice, they should undergo rigorous assessment. One such initial evaluative step has involved the investigation of LLM performance on the United States Medical Licensing Examination [5,6] and board exams in internal medicine [7], ophthalmology [8] and other fields [9–11]. The variability in model accuracy across internal medicine subspecialties [7] underscores the importance of domain-specific evaluations. Such analyses are lacking in cardiology and would serve as a first step to benchmarking LLMs’ fund of knowledge against that of practicing cardiologists.

In this context, comparisons between open-weight and proprietary LLMs are needed, as the former offer enhanced patient data safety, improved bias evaluation, and greater customization potential [12]. Additionally, analyzing the relationship between model size and exam performance is key to estimating the computational resources required for factually accurate medical LLMs. Understanding how model characteristics and retrieval-based techniques impact accuracy is essential for ensuring real-world feasibility. Yet, these relationships remain minimally explored in the cardiovascular literature. In this study, we evaluate open-weight and proprietary LLMs of varying model parameter size, with and without the application of RAG, on cardiology board exam-style questions from the American College of Cardiology Self-Assessment Program (ACCSAP).

## Results

**Fig 1:**
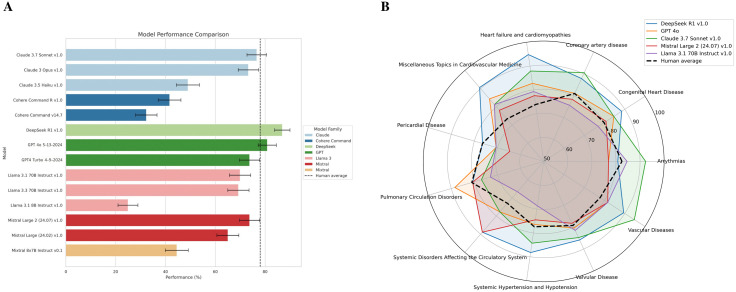
(A) Performance comparison of various LLMs across different model families on cardiology board-style questions, with the mean human performance indicated by a dashed line. (B) Radar chart displaying the percentage of correctly answered cardiology board-style questions by the top-performing LLM from each model family, categorized across major cardiology thematic areas, with the mean human performance indicated by a dashed line.

Both the zero-shot and RAG-based performance of eight proprietary and six open-weight LLMs (**S1 Table**) were evaluated on 449 cardiology board-style questions spanning a broad range of specialty-specific subject areas (**S2 Table**). The models’ zero-shot performance, expressed as a percentage of correct answer choices (accuracy with 95% Wilson confidence intervals), is summarized in **Fig 1A** (**S3 Table**). Focusing on the zero-shot setting, overall accuracy differed significantly across models (Cochran’s Q, *p* < 0.001). The top five zero-shot performing models in descending order include: DeepSeek R1 (86.9%, 95% CI: 83.4-89.7%), GPT 4o (80.9%, 95% CI: 76.9-84.2%), Claude 3.7 Sonnet (76.6%, 95% CI: 72.5-80.3%), GPT 4 Turbo (73.7%, 95% CI: 69.5-77.6%), and Mistral Large 2 (73.7%, 95% CI: 69.5-77.6%). The two highest performing open-weight models included DeepSeek R1 and Mistral Large 2 with 671 and 123 billion parameters, respectively. Exact McNemar tests with Holm correction confirmed that the majority of pairwise differences (79 of 105) were statistically significant (**S3 Table**). Specifically, DeepSeek R1 significantly outperformed GPT-4o by +6.0% (Δ accuracy 95% CI: 3.3, 8.6), *p* < 0.001) and GPT-4o significantly (*p* < 0.01) outperformed Claude 3.7 Sonnet by +4.2%. None of the models in the zero-shot setting demonstrated statistically significant superiority to the human user-derived mean accuracy of 78%, and even DeepSeek R1, the highest-performing model, showed no significant difference under paired per-question McNemar testing (*p* = 0.10). We also assessed OpenEvidence, an LLM-powered proprietary commercial platform designed to deliver clinical answers to healthcare professionals, which achieved a performance of 81.3% (95% CI: 77.4-84.7%) on par (statistically non-significant) with the second highest performing model, GPT 4o. Interestingly, Llama 3.1 8B exhibited a bias towards response “A” resulting in its observed underperformance. Focusing on one top performing LLM per model family, **Fig 1B** depicts model accuracy across key cardiology subject areas. DeepSeek R1 achieved the highest accuracy in 7 of 11 subject areas, followed by Claude 3.7 Sonnet in 3 of 11, and GPT-4o in one.

**Fig 2:**
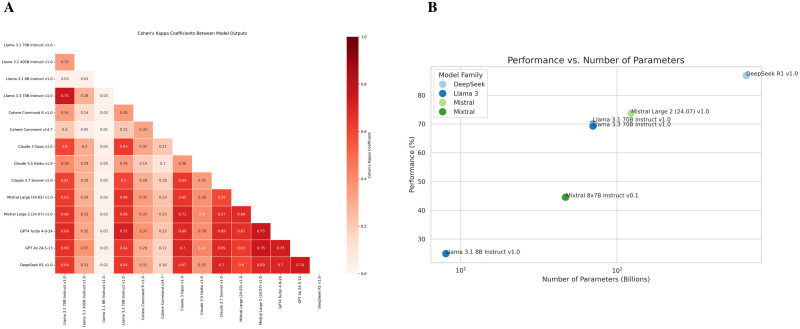
(A) Heatmap showing the pairwise Cohen’s Kappa coefficients for the answer choices of different LLMs on cardiology board-style questions, with red indicating stronger agreement between models. (B) Scatter plot showing the relationship between the log-transformed number of model parameters (in billions) and response accuracy (%) on cardiology board-style questions across different LLMs with publicly available parameter counts.

The highest performing model, DeepSeek R1, exhibited the highest pairwise agreement scores with the other top four performing models, both open-weight and proprietary (**Fig 2A**). The pairwise agreement scores assessed concordance for each question and the highest score achieved was 0.76 between Mistral Large 2 (open-weight) and GPT 4o (proprietary). All remaining scores were lower, indicating a higher degree of variability in the answers each model selected. The percentage of correct answers for each model was graphed against the log-transformed number of model parameters (**Fig 2B**). The observed positive correlation suggests that both across and within model families, increasing the number of model parameters results in improved exam performance (**Fig 2B**).

**Fig 3:**
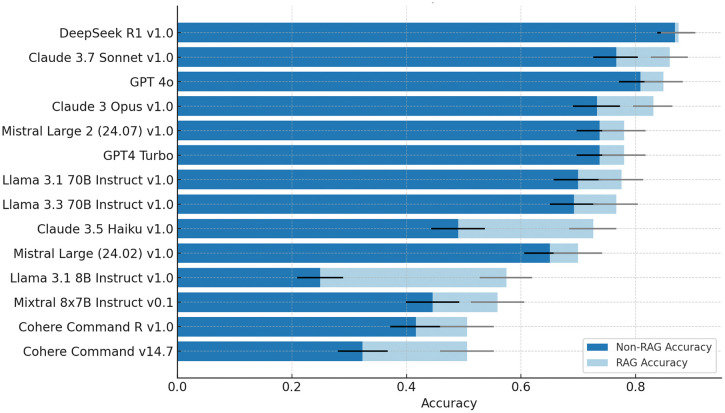
Performance comparison of various LLMs before and after applying RAG in a descending order of accuracy.

Comparison of model performance before and after integrating RAG (**Fig 3**) illustrates that all models showed numerical accuracy improvements following RAG integration, though to varying degrees. After RAG, the top five performing models in descending order are: DeepSeek R1, Claude 3.7 Sonnet, GPT 4o, Claude 3 Opus and Mistral Large 2. Hence the Claude models 3.7 Sonnet and 3 Opus overtook GPT 4o and GPT4 Turbo, respectively. **S4 Table** summarizes the model accuracies after RAG, paired accuracy differences (Δ, percentage points, 95% CI), and results of statistical comparisons (paired exact McNemar tests with Holm correction) for each zero-shot versus RAG model pair. Out of the fourteen models evaluated, nine demonstrated statistically significant accuracy improvements after RAG integration. DeepSeek R1 achieved the smallest absolute (~1.2%) and statistically non-significant improvement with RAG, yet remained the top performing model achieving 88.1% (95% CI: 84.8-90.8%). Among the top five performing models, Claude 3.7 Sonnet and Claude 3 Opus achieved the greatest relative performance improvements at 12.2% and 13.3%, respectively, both of which were statistically significant, resulting in overall accuracies of 86.0% (95% CI: 82.5-88.9%) and 83.1% (95% CI: 79.3-86.3%). GPT 4o and Mistral Large 2 exhibited statistically non-significant and similar absolute performance improvements of +4.0% and +4.3%, achieving post-RAG accuracies of 84.9% (95% CI: 81.2-87.9%) and 78.0% (95% CI: 73.9-81.5%), respectively. Overall, the greatest performance gains were observed among models with the poorest zero-shot performance. In a statistically significant fashion, Llama 3.1 8B improved by 130% (Δ = +32.6 percentage points, accuracy 57.5%, 95% CI: 52.9-62.0%), Cohere Command v14.7 by 56.6% (Δ = +18.3 percentage points, accuracy 50.6%, 95% CI: 45.9-55.2%), and Claude 3.5 Haiku by 48.2% (Δ = +23.6 percentage points, accuracy 72.6%, 95% CI: 68.5-76.7%). Interestingly, the RAG process resolved Llama 3.1 8B’s bias issue towards “A” responses.

## Discussion

In this study, we systematically compared the accuracy of fourteen LLMs from various families, across varying model sizes and degrees of transparency (open-weight versus proprietary), on 449 cardiology board exam-style questions (**Central Illustration**). The recently developed open-weight model DeepSeek R1 outperformed all proprietary alternatives (including OpenAI’s GPT 4o) and led across nearly all cardiology subdomains. While a positive correlation between model size and accuracy was observed within model families, performance varied significantly across families, even among LLMs with similar parameter counts. The use of RAG improved performance across all models, with open-weight models achieving performance levels comparable to proprietary ones.

LLMs are already being incorporated into medical practice [13], despite the lack of an established, systematic evaluation protocol prior to clinical use. A recent publication proposed the development of Artificial Intelligence Structured Clinical Examinations (AI-SCEs) [14]. Emulating Objective Structured Clinical Examinations (OSCEs) used in medical trainees’ assessments, AI-SCEs would involve complex real-world clinical scenarios designed by interdisciplinary teams of clinicians, computer scientists, and medical researchers [14]. Until formal protocols are established, however, medical LLMs should at least demonstrate high factual accuracy on the same medical examinations used to certify physicians [15]. Although this alone is insufficient to establish clinical utility, it is a key first evaluative step. This way, LLM performance can be benchmarked against the existing knowledge base of practicing clinicians. The current work therefore addresses a key gap in the current understanding of LLM competency in cardiovascular medicine.

The leading performance of DeepSeek challenges the prevailing assumption that proprietary models inherently outperform their open-weight counterparts, instead suggesting that large open-weight models may already be capable of rivaling the most advanced commercial systems even in specialized medical knowledge. This is particularly important for the medical field, because open-weight LLMs offer key advantages over proprietary ones. Proprietary LLMs often necessitate uploading private data to online servers and the lack of transparency raises questions about how protected patient information might be stored or used, such as for further training purposes [16]. In contrast, open-weight LLMs can operate offline on secure platforms protecting patient confidentiality by reducing the likelihood of data breaches [12]. Importantly, although these models are not fully open-source, their open-weight nature still enables practical transparency and customization through weight inspection and modification, local fine-tuning, controlled deployment, and independent bias evaluation [12]. While our evaluation focused on general-purpose open-weight LLMs, medically fine-tuned open-source LLMs (e.g., MedAlpaca [17], Me LLaMA [18] or PMC-LLaMA [12]), particularly when coupled with RAG, represent a promising future direction of research.

Despite being positioned as a specialized clinical platform for evidence-based answers, OpenEvidence achieved a score of 81.3%, similar to GPT 4o and lower than DeepSeek R1, both general-purpose LLMs not developed for medical use, with the latter notably being open-weight. This raises important concerns about the added value of proprietary platforms that are already being used in clinical practice and often lack independent external validation. In cardiovascular medicine, this limited assessment suggests that LLM-powered commercial platforms are not necessarily superior to freely accessible open-weight models such as Mistral Large 2 post-RAG or DeepSeek R1. This raises broader questions about whether the restricted access, lack of transparency, and commercial cost associated with these platforms are justified.

Additionally, we demonstrate a trend within LLM families (e.g., Mistral and Llama), where performance in cardiology exam questions improves as parameter size increases. This suggests that, within a single family, larger models generally have better factual accuracy in accordance with prior results [15]. This has important implications for the use of these models in real-world healthcare settings, since the larger the model the greater the computational resource demands. This could limit the accessibility of highly accurate LLMs to smaller healthcare systems, pushing them toward reliance on commercial LLM products or partnerships with larger health systems that have the necessary computational infrastructure. These dynamics risk exacerbating existing disparities in healthcare quality, disproportionately affecting vulnerable populations. Less resourced, more rural, and relatively marginalized healthcare systems may face greater barriers to accessing these advanced tools, further entrenching inequities [19].

At the same time, we also observed considerable differences in performance across different LLM families independent of varying parameter sizes. For instance, Llama 3.1 and 3.3 70b had a comparable performance to Mistral Large 2, despite the latter having an additional 53 billion parameters. This discrepancy indicates that factors beyond the sheer size of a model, such as differences in model architecture, the quality and diversity of training data, and pre-training objectives, have a significant impact on performance. Coupled with our observation that the lowest-performing models (often also the smallest by parameter count) demonstrated the greatest relative improvements after RAG integration, this suggests that smaller, open-weight models can be strategically augmented to close the performance gap. Such gains may also be achievable by focusing fine-tuning efforts on areas of relative deficiency, as identified in our analysis of variability across cardiology subdomains. Notably, RAG also corrected the pronounced answer-choice bias observed in Llama 3.1 8B, highlighting its potential role not only as a performance booster but also as a mechanism for mitigating systematic model-level errors. With targeted retrieval or fine-tuning, these models may achieve accuracy comparable to much larger counterparts without incurring the computational demands or privacy risks associated with proprietary large-scale LLMs. We submit that RAG does introduce inference-time (during each user query) computational overhead, but these costs are largely front-loaded, scale linearly with corpus size, and are typically far lower than those associated with large-scale model training or fine-tuning [20]. Future work is warranted to define the optimal tradeoff of model size (and subsequent resource-use) versus performance, which may vary according to specific intended applications.

The present study’s limitations include the aforementioned inability to test ABIM questions directly and the use of solely text-based questions. This exclusion of image-based questions reflects a constraint of LLMs in their current form and could result in the disproportionate representation of only certain subject areas. Additionally, the absence of a definitive gold-standard measure of human performance is another key limitation. While the rolling average of approximately 78% from ACCSAP provides a useful benchmark, it is derived from a third-party aggregate metric for which individual user characteristics and expertise levels are not accessible to the investigators, and it is influenced by factors such as repeated question exposure and evolving user performance over time. Because the clinical interpretation of absolute LLM performance depends on the true level of expert human performance, shifts in this benchmark affect inferred clinical utility, but relative model ranking, and comparative performance remain unchanged. Moreover, query reformulation in the RAG pipeline was performed by the models themselves, hence relative improvements may partially reflect differences in semantic query construction in addition to downstream evidence integration. Importantly, this behavior was intentionally preserved to reflect standard real-world RAG operation in which upstream models reinterpret retrieval-underspecified queries to optimize semantic retrieval from fixed knowledge bases. Finally, while the identical standardized instructional content was used for all models, OpenAI models required separation of system and user messages due to API access constraints. Although this unavoidable technical difference could theoretically introduce bias, the closed nature of these proprietary models precludes direct mechanistic interrogation of any such effect.

## Conclusion

This study demonstrates that both open and proprietary LLMs can achieve high performance on cardiology board exam-style questions, with the open-weight model DeepSeek R1 outperforming all proprietary alternatives and exceeding the human test-taking benchmark. While model size generally correlated with accuracy within families, cross-family comparisons revealed that parameter count alone does not determine performance, underscoring the influence of model architecture and design. Importantly, RAG substantially boosted the performance of smaller, lower-performing models, and in some cases corrected systemic biases. These findings suggest a promising path forward: smaller, open-weight models, when strategically augmented with retrieval or fine-tuned based on subspecialty-specific deficiencies, may achieve high accuracy without the privacy risks or infrastructure demands of large proprietary systems. Future studies should evaluate the high-performing open-weight models augmented with RAG beyond this initial closed-ended benchmarking on open-ended clinical tasks such as differential diagnosis generation and clinical documentation to better assess real-world clinical utility.

## Materials and methods

### Question selection

Since the American Board of Internal Medicine (ABIM) does not have any publicly available testing material, we used ACCSAP questions that are similar in complexity to the multiple-choice questions from the ABIM cardiovascular disease certification exam. ACCSAP questions cover a wide range of knowledge from diagnosis to medical management and are commonly used to prepare for the ABIM certification exam. ACCSAP reports the average percentage of correct responses by human users for each question. This human test-taking average can change over time, reflecting the cumulative performance of all user responses per question within the most recent iteration of the question bank. As a proprietary database existing behind a paywall, ACCSAP questions are not freely accessible on the internet and should be outside the scope of LLM training data. This was confirmed by a Google index search using the exact first ten words of each question prompt, with the date filter set to before March 15, 2025. All questions containing clinical images were excluded and 449 questions were randomly selected comprising all major subject areas as defined by the ACCSAP, with their percentage distribution included in **S1 Table**. Ethical approval was waived for this study, as it did not involve human participants or identifiable patient data.

### Large language model selection

We evaluated a number of contemporary LLMs, which are listed in **S2 Table** along with their access type, release date, context length, and parameter size (when known). LLM openness refers to the degree to which a model’s components (e.g., architecture, pretrained weights, training code, training datasets) are publicly available under licenses that allow independent use, modification, and sharing. A proprietary model (Claude, GPT, Mistral Large 24.02 v1.0) restricts access to its components, offering use only through an Application Programming Interface (API) or licensed platforms without allowing inspection, modification, or self-hosting. An open weight model (DeepSeek, Llama 3.1/3.3, Mistral Large 24.07 v1.0) publicly releases its pretrained weights for download and local use, but other model components, such as training data/code and data curation methods, are typically withheld, limiting full reproducibility. An open-source model makes all key components publicly available under an open-source license, allowing anyone to use, modify, reproduce, and distribute the model without restriction. Based on the aforementioned definitions, the present study evaluated proprietary and open weight models.

### Inference and hyperparameter settings

Inferences from each model were obtained via SageMaker or Bedrock Amazon Web Services (AWS, Seattle, WA, USA). The LLMs, according to the extent to which each of them could be parameterized, were configured for temperature, top-P and output token limit. Temperature is the parameter responsible for the degree of smoothing of the output token probability distribution. As temperature rises, the probability distribution approximates the uniform distribution, effectively reducing the output token selection to random choice. Top-P, on the other hand, sets a cutoff according to the cumulative probability distribution of the tokens, limiting the tokens to be considered for selection. All experiments were conducted with a temperature of 0, a top-P of 0.9 and a max output token limit of 512 [21]. This combination of hyperparameters was selected to ensure high reproducibility (low temperature), while leaving room for creative expression (high top-P). Setting the temperature to zero ensures deterministic and fully reproducible outputs by always selecting the highest-probability token at each step. The top-P parameter of 0.9 restricts the token selection to the most probable tokens that cumulatively account for 90% of the probability mass, enhancing the coherence and relevance of the generated text [21].

### Performance evaluation

All special characters except for punctuation marks were removed from each ACCSAP question prior to model inference. To ensure that models provided a clear, single answer choice along with a supporting explanation, each question was wrapped into the following prompt: “You are taking an examination that assesses your knowledge in cardiology within the field of medicine. For each question, you answer first with the letter corresponding to the correct answer and then the explanation as to why you have selected the answer. Question: {dataset question}”. For OpenAI models, the identical standardized instructional prompt was placed in the system message and the question itself was passed as the user message to comply with API requirements. For all other models, the same combined prompt text was passed as a single input, such that the semantic prompt content, formatting constraints, and answer instructions were identical across models. After inference, model outputs were reviewed to identify the selected answer choice for each question and calculate each model’s test taking accuracy.

### Retrieval-augmented generation

Retrieval-Augmented Generation (RAG) is a technique that attempts to improve language model performance by retrieving and incorporating relevant content from an external, provided document set during the response generation process. The impact of RAG on the aforementioned model performance was investigated. The entire RAG process for all models was based on Amazon’s Bedrock Knowledge Base service. A total of 123 documents were used, including 14 textbooks (primarily companions to *Braunwald’s Heart Disease*) and 109 cardiology guidelines and expert consensus documents from the American College of Cardiology and European Society of Cardiology from 2012 to 2024 (**S5 Table**). The documents were divided into smaller segments (chunks) following a fixed-size chunking approach. The maximum number of tokens allowed per chunk during document segmentation was set to 500 with an overlap percentage of 20%. The resulted chunks were converted into a vector representation through the amazon.titan-embed-text-v1 embedding model and stored in Amazon OpenSearch. In order to improve the retrieval of relevant chunks, each multiple-choice question was reformulated by each LLM based on the orchestration prompt template (**S1 Fig**). Query reformulation was performed by each model solely to optimize semantic retrieval from a fixed, shared knowledge base, without access to ground-truth labels, reflecting standard real-world RAG deployment. The reformulated question was then transformed into a vector representation using the same embedding model and compared to all chunks following a dynamically adjusted approach (hybrid/semantic). The maximum number of retrieved chunks was set to 15. These chunks where then provided to the generation prompt template (**S2 Fig**), which produced the final prompt that was submitted to the LLM in order to produce the final response.

### Statistical analysis

Model performance was quantified as accuracy, defined as the proportion of questions answered correctly. Ninety-five percent confidence intervals (95% CIs) for accuracy were computed using the Wilson score method for binomial proportions. Because all models were evaluated on the same questions, between-model comparisons were treated as paired outcomes. Overall accuracy differences across models were first assessed using Cochran’s Q test. Post-hoc pairwise comparisons were then performed using exact McNemar tests based on discordant response pairs (one model correct, the other incorrect), with family-wise error controlled using Holm correction at α = 0.05. For paired comparisons, effect sizes were summarized as accuracy differences (Δ, percentage points). For zero-shot versus RAG comparisons, 95% CIs for Δ were obtained using a paired bootstrap over questions (resampling with replacement). Pairwise model agreement was evaluated using Cohen’s κ, computed on binary correctness (correct vs. incorrect relative to the reference answer). All analyses were performed in Python (version 3.2).

## Supporting information

S1 Fig Orchestration prompt template provided to the Large Language Models for the retrieval of relevant text from the supplied documents as part of the Retrieval-Augmented Generation process. (DOCX)

S2 Fig Generation prompt template provided to the Large Language Models for them to answer each question assisted by the Retrieval-Augmentation Generation process. (DOCX)

S1 Table Cardiology topics defined by the American College of Cardiology Self-Assessment Program (ACCSAP), listed in descending order by their percentage composition of the 450 questions used to evaluate Large Language Model (LLM) performance. (DOCX)

S2 Table Overview of access types and model specifications for the investigated Large Language Models (LLMs). (DOCX)

S3 Table Ranked performance of LLMs across zero-shot and RAG settings, showing accuracy (%, 95% Wilson confidence intervals) and the number of pairwise significant wins and losses versus other models under Holm-adjusted exact McNemar testing. (DOCX)

S4 Table Zero-shot versus RAG paired accuracy differences for each model, ranked in descending order of absolute accuracy improvement (Δ, % points). For each model, Δ is reported with its 95% Wilson confidence intervals, together with the exact McNemar *p*-value and the Holm-adjusted *p*-value across all model comparisons. Statistical significance was evaluated using the Holm-adjusted p-value (α = 0.05).
(DOCX)

S5 Table List of societal guidelines/consensus documents and cardiology textbooks in chronological order, which were included in the Knowledge Base as part of the Retrieval-Augmented Generation process. (DOCX)
