# Supplementary material for: Cardiology knowledge assessment of retrieval-augmented open versus proprietary large language models
Source: PLOS Digit Health. 2026 Mar 12;5(3):e0001029. doi: 10.1371/journal.pdig.0001029 (PMC12981508; doi:10.1371/journal.pdig.0001029)
Supplement: S1 Fig — (DOCX) [file pdig.0001029.s001.docx]

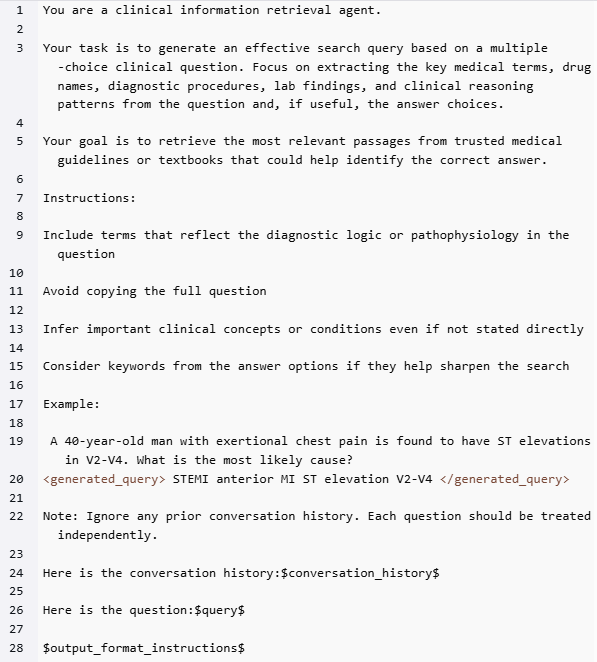


**S1 Fig.** Orchestration prompt template provided to the Large Language Models for the retrieval of relevant text from the supplied documents as part of the Retrieval-Augmented Generation process.
