# Supplementary material for: Cardiology knowledge assessment of retrieval-augmented open versus proprietary large language models
Source: PLOS Digit Health. 2026 Mar 12;5(3):e0001029. doi: 10.1371/journal.pdig.0001029 (PMC12981508; doi:10.1371/journal.pdig.0001029)
Supplement: S2 Fig — (DOCX) [file pdig.0001029.s002.docx]

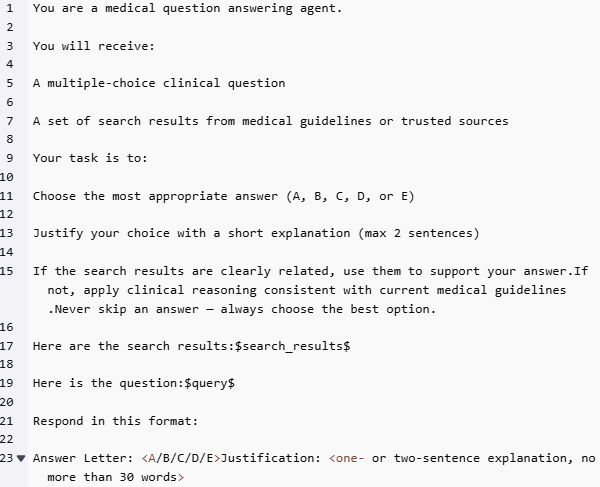


**S2 Fig.** Generation prompt template provided to the Large Language Models for them to answer each question assisted by the Retrieval-Augmentation Generation process.
