# Supplementary material for: Cardiology knowledge assessment of retrieval-augmented open versus proprietary large language models
Source: PLOS Digit Health. 2026 Mar 12;5(3):e0001029. doi: 10.1371/journal.pdig.0001029 (PMC12981508; doi:10.1371/journal.pdig.0001029)
Supplement: S1 Table — (DOCX) [file pdig.0001029.s003.docx]

| **Access Type** | **Company** | **Models series** | **Model name** | **Context window (thousand tokens)** | **Model size (billion parameters)** | **Release date (checkpoint)** |
| --- | --- | --- | --- | --- | --- | --- |
| Proprietary | Cohere | Cohere Command | Cohere Command v14.7 | 4000 | Unknown | 2023-Sep |
|  |  |  | Cohere Command R v1.0 | 128 | Unknown | 2024-Apr-29 |
|  | Anthropic | Claude | Claude 3.5 Haiku v1.0 | 200 | Unknown | 2024-Oct-22 |
|  |  |  | Claude 3 Opus v1.0 | 200 | Unknown | 2024-Apr-16 |
|  |  |  | Claude 3.7 Sonnet v1.0 | 200 | Unknown | 2025-Feb-19 |
|  | OpenAI | GPT | GPT 4o |  | Unknown | 2024-May-13 |
|  |  |  | GPT 4 Turbo |  | Unknown | 2024-Apr-09 |
|  | Mistral AI | Mistral | Mistral Large (24.02) v1.0 | 200 | Unknown | 2024-Feb-02 |
| Open weight |  |  | Mistral Large 2 (24.07) v1.0 | 128 | 123 | 2024-Jul-30 |
|  |  | Mixtral | Mixtral 8x7b Instruct v0.1 | 32 | 47 | 2024-Mar-01 |
|  | Meta | Llama 3.1 | Llama 3.1 8b Instruct v1.0 | 128 | 8 | 2024-Jul-23 |
|  |  |  | Llama 3.1 70b Instruct v1.0 | 128 | 70 | 2024-Jul-23 |
|  |  | Llama 3.3 | Llama 3.3 70B Instruct v1.0 | 128 | 70 | 2024-Dec-18 |
|  | DeepSeek | DeepSeek | DeepSeek R1 | 128 | 671 | 2025-Mar-03 |

**S1 Table.** Overview of access types and model specifications for the investigated Large Language Models (LLMs).
