# Supplementary material for: Cardiology knowledge assessment of retrieval-augmented open versus proprietary large language models
Source: PLOS Digit Health. 2026 Mar 12;5(3):e0001029. doi: 10.1371/journal.pdig.0001029 (PMC12981508; doi:10.1371/journal.pdig.0001029)
Supplement: S2 Table — (DOCX) [file pdig.0001029.s004.docx]

| **Cardiology topic** | **Question bank percentage (%)** |
| --- | --- |
| Valvular disease | 20 |
| Coronary artery disease | 17 |
| Heart failure and cardiomyopathies | 13 |
| Systemic hypertension and hypotension | 11 |
| Vascular diseases | 8 |
| Miscellaneous topics in cardiovascular medicine | 7 |
| Arrhythmias | 6 |
| Congenital heart disease | 6 |
| Pulmonary circulation disorder | 6 |
| Pericardial disease | 4 |
| Systemic disorders affecting the circulatory system | 2 |

**S2 Table.** Cardiology topics defined by the American College of Cardiology Self-Assessment Program (ACCSAP), listed in descending order by their percentage composition of the 450 questions used to evaluate Large Language Model (LLM) performance.
