# Supplementary material for: Cardiology knowledge assessment of retrieval-augmented open versus proprietary large language models
Source: PLOS Digit Health. 2026 Mar 12;5(3):e0001029. doi: 10.1371/journal.pdig.0001029 (PMC12981508; doi:10.1371/journal.pdig.0001029)
Supplement: S3 Table — (DOCX) [file pdig.0001029.s005.docx]

| **Rank** | **Model** | **Setting** | **Accuracy (%)** | **95% confidence intervals** | **Significant wins (McNemar)** | **Significant losses (McNemar)** |
| --- | --- | --- | --- | --- | --- | --- |
| 1 | DeepSeek R1 v1.0 | RAG | 88.1 | 84.8, 90.8 | 24 | 0 |
| 2 | DeepSeek R1 v1.0 | Zero-shot | 86.9 | 83.4, 89.7 | 23 | 0 |
| 3 | Claude 3.7 Sonnet v1.0 | RAG | 86.0 | 82.5, 88.9 | 23 | 0 |
| 4 | GPT 4o | RAG | 84.9 | 81.2, 87.9 | 22 | 0 |
| 5 | Claude 3 Opus v1.0 | RAG | 83.1 | 79.3, 86.3 | 18 | 0 |
| 6 | OpenEvidence | Zero-shot | 81.3 | 77.4, 84.7 | 18 | 1 |
| 7 | GPT 4o | Zero-shot | 80.9 | 77.0, 84.2 | 15 | 0 |
| 8 | GPT4 Turbo | RAG | 78.0 | 73.9, 81.5 | 13 | 5 |
| 9 | Mistral Large 2 (24.07) v1.0 | RAG | 78.0 | 73.9, 81.5 | 13 | 4 |
| 10 | Llama 3.1 70B Instruct v1.0 | RAG | 77.5 | 73.4, 81.1 | 13 | 5 |
| 11 | Llama 3.3 70B Instruct v1.0 | RAG | 76.6 | 72.5, 80.3 | 11 | 5 |
| 12 | Claude 3.7 Sonnet v1.0 | Zero-shot | 76.6 | 72.5, 80.3 | 11 | 5 |
| 13 | GPT4 Turbo | Zero-shot | 73.7 | 69.5, 77.6 | 11 | 7 |
| 14 | Mistral Large 2 (24.07) v1.0 | Zero-shot | 73.7 | 69.5, 77.6 | 11 | 8 |
| 15 | Claude 3 Opus v1.0 | Zero-shot | 73.3 | 69.0, 77.2 | 10 | 7 |
| 16 | Claude 3.5 Haiku v1.0 | RAG | 72.6 | 68.3, 76.5 | 10 | 7 |
| 17 | Mistral Large (24.02) v1.0 | RAG | 69.9 | 65.5, 74.0 | 10 | 10 |
| 18 | Llama 3.1 70B Instruct v1.0 | Zero-shot | 69.9 | 65.5, 74.0 | 10 | 9 |
| 19 | Llama 3.3 70B Instruct v1.0 | Zero-shot | 69.3 | 64.9, 73.4 | 10 | 11 |
| 20 | Mistral Large (24.02) v1.0 | Zero-shot | 65.3 | 60.7, 69.5 | 8 | 15 |
| 21 | Llama 3.1 8B Instruct v1.0 | RAG | 57.5 | 52.8, 62.0 | 5 | 20 |
| 22 | Mixtral 8x7B Instruct v0.1 | RAG | 55.9 | 51.3, 60.4 | 5 | 20 |
| 23 | Cohere Command R v1.0 | RAG | 50.6 | 46.0, 55.2 | 3 | 21 |
| 24 | Cohere Command v14.7 | RAG | 50.6 | 46.0, 55.2 | 3 | 21 |
| 25 | Claude 3.5 Haiku v1.0 | Zero-shot | 49.0 | 44.4, 53.6 | 2 | 21 |
| 26 | Mixtral 8x7B Instruct v0.1 | Zero-shot | 44.5 | 40.0, 49.2 | 2 | 23 |
| 27 | Cohere Command R v1.0 | Zero-shot | 41.7 | 37.2, 46.3 | 1 | 23 |
| 28 | Cohere Command v14.7 | Zero-shot | 32.3 | 28.1, 36.8 | 0 | 27 |
| 29 | Llama 3.1 8B Instruct v1.0 | Zero-shot | 24.9 | 21.2, 29.2 | 0 | 29 |

**S3 Table.** Ranked performance of LLMs across zero-shot and RAG settings, showing accuracy (%, 95% Wilson confidence intervals) and the number of pairwise significant wins and losses versus other models under Holm-adjusted exact McNemar testing.
