# Supplementary material for: Cardiology knowledge assessment of retrieval-augmented open versus proprietary large language models
Source: PLOS Digit Health. 2026 Mar 12;5(3):e0001029. doi: 10.1371/journal.pdig.0001029 (PMC12981508; doi:10.1371/journal.pdig.0001029)
Supplement: S4 Table — For each model, Δ is reported with its 95% Wilson confidence intervals, together with the exact McNemar p-value and the Holm-adjusted p-value across all model comparisons. Statistical significance was evaluated using the Holm-adjusted p-value (α = 0.05). (DOCX) [file pdig.0001029.s006.docx]

| **RAG-Model** | **Zero-shot versus RAG ∆ (%)** | **∆ 95% CI** | **Exact McNemar**  ***p*-value** | **Holm-adjusted**  ***p*-value** |
| --- | --- | --- | --- | --- |
| Llama 3.1 8B Instruct v1.0 | 32.6 | 26.3, 38.3 | <0.001 | <0.001 |
| Claude 3.5 Haiku v1.0 | 23.6 | 18.7, 28.5 | <0.001 | <0.001 |
| Cohere Command v14.7 | 18.3 | 12.7, 23.6 | <0.001 | <0.001 |
| Mixtral 8x7B Instruct v0.1 | 11.4 | 6.5, 16.5 | <0.001 | <0.001 |
| Claude 3 Opus v1.0 | 9.8 | 6.0, 13.6 | <0.001 | <0.001 |
| Claude 3.7 Sonnet v1.0 | 9.4 | 5.6, 13.4 | <0.001 | <0.001 |
| Cohere Command R v1.0 | 8.9 | 4.0, 13.8 | <0.001 | 0.003 |
| Llama 3.1 70B Instruct v1.0 | 7.6 | 3.6, 11.6 | <0.001 | 0.003 |
| Llama 3.3 70B Instruct v1.0 | 7.3 | 3.3, 11.4 | <0.001 | 0.003 |
| Mistral Large (24.02) v1.0 | 4.6 | 1.1, 8.5 | 0.02 | 0.09 |
| GPT4 Turbo | 4.3 | 1.1, 7.8 | 0.02 | 0.09 |
| Mistral Large 2 (24.07) v1.0 | 4.3 | 0.4, 8.2 | 0.05 | 0.10 |
| GPT 4o | 4.0 | 0.5, 7.4 | 0.02 | 0.09 |
| DeepSeek R1 v1.0 | 1.2 | -2.2, 3.8 | 0.67 | 0.67 |

**S4 Table.** Zero-shot versus RAG paired accuracy differences for each model, ranked in descending order of absolute accuracy improvement (Δ, % points). For each model, Δ is reported with its 95% Wilson confidence intervals, together with the exact McNemar p-value and the Holm-adjusted p-value across all model comparisons. Statistical significance was evaluated using the Holm-adjusted p-value (α = 0.05).
