# Supplementary material for: Cardiology knowledge assessment of retrieval-augmented open versus proprietary large language models
Source: PLOS Digit Health. 2026 Mar 12;5(3):e0001029. doi: 10.1371/journal.pdig.0001029 (PMC12981508; doi:10.1371/journal.pdig.0001029)
Supplement: S5 Table — (DOCX) [file pdig.0001029.s007.docx]

| **No.** | **Title** | **Type** |
| --- | --- | --- |
| 1 | Newby, L.K. *et al.* 2012 Expert Consensus Document on the management of patients with ischemic heart disease and diabetes mellitus. *J. Am. Coll. Cardiol.* **60**, 2287–2302 (2012). | ACC expert consensus |
| 2 | O'Gara, P.T. *et al.* 2013 ACCF/AHA guideline for the management of ST-elevation myocardial infarction: a report of the American College of Cardiology Foundation/American Heart Association Task Force on Practice Guidelines. *J. Am. Coll. Cardiol.* **61**, e78–e140 (2013). | ACC Guidelines |
| 3 | Kusumoto, F.M. *et al.* HRS/ACC/AHA Expert Consensus Statement on the use of implantable cardioverter-defibrillator therapy in patients who are not included or not well represented in clinical trials. *J. Am. Coll. Cardiol.* 64, 1115–1133 (2014). | ACC expert consensus |
| 4 | Maron, B.J. *et al.* Assessment of the 12-lead electrocardiogram as a screening test for detection of cardiovascular disease in healthy general populations of young people (12–25 years of age): a scientific statement from the American Heart Association and the American College of Cardiology. *J. Am. Coll. Cardiol.* **64**, 1479–1514 (2014). | ACC expert consensus |
| 5 | Amsterdam, E.A. *et al.* 2014 AHA/ACC guideline for the management of patients with non–ST-elevation acute coronary syndromes: a report of the American College of Cardiology/American Heart Association Task Force on Practice Guidelines. *J. Am. Coll. Cardiol.* **64**, e139–e228 (2014). | ACC Guidelines |
| 6 | Fleisher, L.A. *et al.* 2014 ACC/AHA guideline on perioperative cardiovascular evaluation and management of patients undergoing noncardiac surgery: a report of the American College of Cardiology/American Heart Association Task Force on Practice Guidelines. *J. Am. Coll. Cardiol.* **64**, e77–e137 (2014). | ACC Guidelines |
| 7 | Erbel, R. *et al.* 2014 ESC Guidelines on the diagnosis and treatment of aortic diseases: developed by the Task Force of the European Society of Cardiology (ESC). *Eur. Heart J.* **35**, 2873–2926 (2014). | ESC Guidelines |
| 8 | Maron, B.J. *et al.* Eligibility and disqualification recommendations for competitive athletes with cardiovascular abnormalities: preamble, principles, and general considerations: a scientific statement from the American Heart Association and American College of Cardiology. *J. Am. Coll. Cardiol.* **66**, 2343–2349 (2015). | ACC expert consensus |
| 9 | Rihal, C.S. *et al.* 2015 SCAI/ACC/HFSA/STS Clinical Expert Consensus Statement on the use of percutaneous mechanical circulatory support devices in cardiovascular care. *J. Am. Coll. Cardiol.* **65**, 1662–1672 (2015). | ACC expert consensus |
| 10 | Page, R.L. *et al.* 2015 ACC/AHA/HRS Guideline for the management of adult patients with supraventricular tachycardia: a report of the American College of Cardiology/American Heart Association Task Force on Clinical Practice Guidelines and the Heart Rhythm Society. *J. Am. Coll. Cardiol.* **67**, e1–e72 (2016). | ACC Guidelines |
| 11 | Adler, Y. *et al.* 2015 ESC Guidelines for the diagnosis and management of pericardial diseases: developed by the Task Force of the European Society of Cardiology (ESC). *Eur. Heart J.* **36**, 2921–2964 (2015). | ESC Guidelines |
| 12 | Levine, G.N. *et al.* 2016 ACC/AHA Guideline focused update on duration of dual antiplatelet therapy in patients with coronary artery disease: a report of the American College of Cardiology/American Heart Association Task Force on Clinical Practice Guidelines. *J. Am. Coll. Cardiol.* **68**, 1082–1115 (2016). | ACC Guidelines |
| 13 | Rich, M.W. *et al.* Knowledge gaps in cardiovascular care of the older adult population: a scientific statement from the American Heart Association, American College of Cardiology, and American Geriatrics Society. *J. Am. Coll. Cardiol.* **67**, 730–738 (2016). | ACC expert consensus |
| 14 | Kavinsky, C.J. *et al.* SCAI/ACC/HRS institutional and operator requirements for left atrial appendage occlusion. *J. Am. Coll. Cardiol.* **67**, 2362–2375 (2016). | ACC expert consensus |
| 15 | Lloyd-Jones, D.M. *et al.* 2016 ACC Expert Consensus Decision Pathway on the role of non-statin therapies for LDL-cholesterol lowering in the management of atherosclerotic cardiovascular disease risk: a report of the American College of Cardiology Task Force on Clinical Expert Consensus Documents. *J. Am. Coll. Cardiol.* **68**, 2504–2520 (2016). | ACC expert consensus |
| 16 | Gerhard-Herman, M.D. *et al.* 2016 AHA/ACC Guideline on the management of patients with lower extremity peripheral artery disease: a report of the American College of Cardiology/American Heart Association Task Force on Clinical Practice Guidelines. *J. Am. Coll. Cardiol.* **69**, e71–e126 (2017). | ACC Guidelines |
| 17 | Doherty, J.U. *et al.* 2017 ACC Expert Consensus Decision Pathway for periprocedural management of anticoagulation in patients with nonvalvular atrial fibrillation: a report of the American College of Cardiology Clinical Expert Consensus Document Task Force. *J. Am. Coll. Cardiol.* **69**, 3027–3058 (2017). | ACC expert consensus |
| 18 | Tomaselli, G.F. *et al.* 2017 ACC Expert Consensus Decision Pathway on management of bleeding in patients on oral anticoagulants: a report of the American College of Cardiology Task Force on Expert Consensus Decision Pathways. *J. Am. Coll. Cardiol.* **70**, 2077–2109 (2017). | ACC expert consensus |
| 19 | Yancy, C.W. *et al.* 2017 ACC Expert Consensus Decision Pathway for optimization of heart failure treatment: answers to 10 pivotal issues about heart failure with reduced ejection fraction: a report of the American College of Cardiology Task Force on Expert Consensus Decision Pathways. *J. Am. Coll. Cardiol.* **71**, 201–230 (2018). | ACC expert consensus |
| 20 | O'Gara, P.T. *et al.* 2017 ACC Expert Consensus Decision Pathway on the management of mitral regurgitation: a report of the American College of Cardiology Task Force on Expert Consensus Decision Pathways. *J. Am. Coll. Cardiol.* **70**, 2421–2447 (2017). | ACC expert consensus |
| 21 | Otto, C.M. *et al.* 2017 ACC Expert Consensus Decision Pathway for transcatheter aortic valve replacement in the management of adults with aortic stenosis: a report of the American College of Cardiology Task Force on Clinical Expert Consensus Documents. *J. Am. Coll. Cardiol.* **69**, 1313–1346 (2017). | ACC expert consensus |
| 22 | Shen, W.-K. *et al.* 2017 ACC/AHA/HRS Guideline for the evaluation and management of patients with syncope: a report of the American College of Cardiology/American Heart Association Task Force on Clinical Practice Guidelines and the Heart Rhythm Society. *J. Am. Coll. Cardiol.* **70**, e39–e110 (2017). | ACC Guidelines |
| 23 | Al-Khatib, S.M. *et al.* 2017 AHA/ACC/HRS Guideline for the management of patients with ventricular arrhythmias and the prevention of sudden cardiac death: a report of the American College of Cardiology/American Heart Association Task Force on Clinical Practice Guidelines and the Heart Rhythm Society. *J. Am. Coll. Cardiol.* **72**, e91–e220 (2018). | ACC Guidelines |
| 24 | Valgimigli, M. *et al.* 2017 ESC focused update on dual antiplatelet therapy in coronary artery disease: developed in collaboration with EACTS. *Eur. Heart J.* **39**, 213–254 (2018). | ESC Guidelines |
| 25 | Aboyans, V. *et al.* 2017 ESC Guidelines on the diagnosis and treatment of peripheral arterial diseases, in collaboration with the European Society for Vascular Surgery (ESVS): developed by the Task Force of the European Society of Cardiology (ESC) and the European Society for Vascular Surgery (ESVS). *Eur. Heart J.* 39, 763–821 (2018). | ESC Guidelines |
| 26 | Ibanez, B. *et al.* 2017 ESC Guidelines for the management of acute myocardial infarction in patients presenting with ST-segment elevation: developed by the Task Force of the European Society of Cardiology (ESC). *Eur. Heart J.* **39**, 119–177 (2018). | ESC Guidelines |
| 27 | Whelton, P.K. *et al.* 2017 ACC/AHA/AAPA/ABC/ACPM/AGS/APhA/ASH/ASPC/NMA/PCNA Guideline for the prevention, detection, evaluation, and management of high blood pressure in adults: a report of the American College of Cardiology/American Heart Association Task Force on Clinical Practice Guidelines. *J. Am. Coll. Cardiol.* **71**, e13–e115 (2018). | ACC Guidelines |
| 28 | Kusumoto, F.M. *et al.* 2018 ACC/AHA/HRS Guideline on the evaluation and management of patients with bradycardia and cardiac conduction delay: a report of the American College of Cardiology/American Heart Association Task Force on Clinical Practice Guidelines and the Heart Rhythm Society. *J. Am. Coll. Cardiol.* **74**, e51–e156 (2019). | ACC Guidelines |
| 29 | Grundy, S.M. *et al.* 2018 AHA/ACC/AACVPR/AAPA/ABC/ACPM/ADA/AGS/APhA/ASPC/NLA/PCNA Guideline on the management of blood cholesterol: a report of the American College of Cardiology/American Heart Association Task Force on Clinical Practice Guidelines. *J. Am. Coll. Cardiol.* **73**, e285–e350 (2019). | ACC Guidelines |
| 30 | Stout, K.K. *et al.* 2018 AHA/ACC Guideline for the management of adults with congenital heart disease: a report of the American College of Cardiology/American Heart Association Task Force on Clinical Practice Guidelines. *J. Am. Coll. Cardiol.* **73**, e81–e192 (2019). | ACC Guidelines |
| 31 | Das, S.R. *et al.* 2018 ACC Expert Consensus Decision Pathway on novel therapies for cardiovascular risk reduction in patients with type 2 diabetes and atherosclerotic cardiovascular disease: a report of the American College of Cardiology Task Force on Expert Consensus Decision Pathways. *J. Am. Coll. Cardiol.* **72**, 3310–3342 (2018). | ACC expert consensus |
| 32 | Hirshfeld, J.W. Jr. *et al.* 2018 ACC/HRS/NASCI/SCAI/SCCT Expert Consensus Document on optimal use of ionizing radiation in cardiovascular imaging: best practices for safety and effectiveness. *J. Am. Coll. Cardiol.* **71**, 2829–2845 (2018). | ACC expert consensus |
| 33 | Thygesen, K. *et al.* Fourth universal definition of myocardial infarction (2018): a consensus document of the Joint European Society of Cardiology (ESC)/American College of Cardiology (ACC)/American Heart Association (AHA)/World Heart Federation (WHF) Task Force. *J. Am. Coll. Cardiol.* **72**, 2231–2264 (2018). | ACC expert consensus |
| 34 | Bavaria, J.E. *et al.* 2018 AATS/ACC/SCAI/STS Expert Consensus Systems of Care Document: operator and institutional recommendations and requirements for transcatheter aortic valve replacement. *J. Am. Coll. Cardiol.* **73**, 340–374 (2019). | ACC expert consensus |
| 35 | Barua, R.S. *et al.* 2018 ACC Expert Consensus Decision Pathway on tobacco cessation treatment: a report of the American College of Cardiology Task Force on Clinical Expert Consensus Documents. *J. Am. Coll. Cardiol.* **72**, 3332–3365 (2018). | ACC expert consensus |
| 36 | Williams, B. *et al.* 2018 ESC/ESH Guidelines for the management of arterial hypertension: developed by the Task Force of the European Society of Cardiology (ESC) and the European Society of Hypertension (ESH). *Eur. Heart J.* **39**, 3021–3104 (2018). | ESC Guidelines |
| 37 | Regitz-Zagrosek, V. *et al.* 2018 ESC Guidelines for the management of cardiovascular diseases during pregnancy: developed by the Task Force of the European Society of Cardiology (ESC) and endorsed by the International Society of Gender Medicine (IGM), the German Institute of Gender in Medicine (DGesGM), the European Society of Anaesthesiology (ESA), and the European Society of Gynecology (ESG). *Eur. Heart J.* **39**, 3165–3241 (2018). | ESC Guidelines |
| 38 | Thygesen, K. *et al.* Fourth universal definition of myocardial infarction (2018): a consensus document of the Joint European Society of Cardiology (ESC)/American College of Cardiology (ACC)/American Heart Association (AHA)/World Heart Federation (WHF) Task Force. *Eur. Heart J.* **40**, 237–269 (2019). | ESC Guidelines |
| 39 | Brignole, M. *et al.* 2018 ESC Guidelines for the diagnosis and management of syncope: developed by the Task Force of the European Society of Cardiology (ESC) with the special contribution of the European Heart Rhythm Association (EHRA). *Eur. Heart J.* **39**, 1883–1948 (2018). | ESC Guidelines |
| 40 | Neumann, F.-J. *et al.* 2018 ESC/EACTS Guidelines on myocardial revascularization: developed by the Task Force of the European Society of Cardiology (ESC) and the European Association for Cardio-Thoracic Surgery (EACTS) with the special contribution of the European Association for Percutaneous Cardiovascular Interventions (EAPCI). *Eur. Heart J.* **40**, 87–165 (2019). | ESC Guidelines |
| 41 | Thomas, R.J. *et al.* Home-based cardiac rehabilitation: a scientific statement from the American Association of Cardiovascular and Pulmonary Rehabilitation, the American Heart Association, and the American College of Cardiology. *J. Am. Coll. Cardiol.* **74**, 133–153 (2019). | ACC expert consensus |
| 42 | Hollenberg, S.M. *et al.* 2019 ACC Expert Consensus Decision Pathway on risk assessment, management, and clinical trajectory of patients hospitalized with heart failure: a report of the American College of Cardiology Solution Set Oversight Committee. *J. Am. Coll. Cardiol.* **74**, 1966–2011 (2019). | ACC expert consensus |
| 43 | Bonow, R.O. *et al.* 2019 AATS/ACC/SCAI/STS Expert Consensus Systems of Care Document: operator and institutional recommendations and requirements for transcatheter mitral valve intervention. *J. Am. Coll. Cardiol.* **76**, 96–117 (2020). | ACC expert consensus |
| 44 | Nishimura, R.A. *et al.* 2019 AATS/ACC/ASE/SCAI/STS Expert Consensus Systems of Care Document: a proposal to optimize care for patients with valvular heart disease. *J. Am. Coll. Cardiol.* **73**, 2609–2635 (2019). | ACC expert consensus |
| 45 | Arnett, D.K. *et al.* 2019 ACC/AHA Guideline on the primary prevention of cardiovascular disease: a report of the American College of Cardiology/American Heart Association Task Force on Clinical Practice Guidelines. *J. Am. Coll. Cardiol.* **74**, e177–e232 (2019). | ACC Guidelines |
| 46 | Konstantinides, S.V. *et al.* 2019 ESC Guidelines for the diagnosis and management of acute pulmonary embolism: developed in collaboration with the European Respiratory Society (ERS). *Eur. Heart J.* **41**, 543–603 (2020). | ESC Guidelines |
| 47 | Knuuti, J. *et al.* 2019 ESC Guidelines for the diagnosis and management of chronic coronary syndromes: developed by the Task Force of the European Society of Cardiology (ESC). *Eur. Heart J.* **41**, 407–477 (2020). | ESC Guidelines |
| 48 | Mach, F. *et al.* 2019 ESC/EAS Guidelines for the management of dyslipidaemias: lipid modification to reduce cardiovascular risk: developed by the Task Force of the European Society of Cardiology (ESC) and the European Atherosclerosis Society (EAS). *Eur. Heart J.* **41**, 111–188 (2020). | ESC Guidelines |
| 49 | Brugada, J. *et al.* 2019 ESC Guidelines for the management of patients with supraventricular tachycardia: developed by the Task Force of the European Society of Cardiology (ESC) in collaboration with the Association for European Paediatric and Congenital Cardiology (AEPC). *Eur. Heart J.* **41**, 655–720 (2020). | ESC Guidelines |
| 50 | Tomaselli, G.F. *et al.* 2020 ACC Expert Consensus Decision Pathway on management of bleeding in patients on oral anticoagulants: a report of the American College of Cardiology Solution Set Oversight Committee. *J. Am. Coll. Cardiol.* **76**, 594–622 (2020). | ACC expert consensus |
| 51 | Lilly, S.M. *et al.* 2020 ACC Expert Consensus Decision Pathway on management of conduction disturbances in patients undergoing transcatheter aortic valve replacement: a report of the American College of Cardiology Solution Set Oversight Committee. *J. Am. Coll. Cardiol.* **76**, 2391–2411 (2020). | ACC expert consensus |
| 52 | Das, S.R. *et al.* 2020 ACC Expert Consensus Decision Pathway on novel therapies for cardiovascular risk reduction in patients with type 2 diabetes: a report of the American College of Cardiology Solution Set Oversight Committee. *J. Am. Coll. Cardiol.* **76**, 1117–1145 (2020). | ACC expert consensus |
| 53 | Bonow, R.O. *et al.* 2020 Focused Update of the 2017 ACC Expert Consensus Decision Pathway on the management of mitral regurgitation: a report of the American College of Cardiology Solution Set Oversight Committee. *J. Am. Coll. Cardiol.* **75**, 2236–2270 (2020). | ACC expert consensus |
| 54 | Ommen, S.R. *et al.* 2020 AHA/ACC Guideline for the diagnosis and treatment of patients with hypertrophic cardiomyopathy: a report of the American College of Cardiology/American Heart Association Joint Committee on Clinical Practice Guidelines. *J. Am. Coll. Cardiol.* **76**, e159–e240 (2020). | ACC Guidelines |
| 55 | Otto, C.M. *et al.* 2020 ACC/AHA Guideline for the management of patients with valvular heart disease: a report of the American College of Cardiology/American Heart Association Joint Committee on Clinical Practice Guidelines. *J. Am. Coll. Cardiol.* **77**, e25–e197 (2021). | ACC Guidelines |
| 56 | Hindricks, G. *et al.* 2020 ESC Guidelines for the diagnosis and management of atrial fibrillation: developed in collaboration with the European Association for Cardio-Thoracic Surgery (EACTS) and with the special contribution of the European Heart Rhythm Association (EHRA) of the ESC. *Eur. Heart J.* **42**, 373–498 (2021). | ESC Guidelines |
| 57 | Baumgartner, H. *et al.* 2020 ESC Guidelines for the management of adult congenital heart disease: developed by the Task Force of the European Society of Cardiology (ESC) and endorsed by the Association for European Paediatric and Congenital Cardiology (AEPC) and the International Society for Adult Congenital Heart Disease (ISACHD). *Eur. Heart J.* **42**, 563–645 (2021). | ESC Guidelines |
| 58 | Pelliccia, A. *et al.* 2020 ESC Guidelines on sports cardiology and exercise in patients with cardiovascular disease: developed by the Task Force of the European Society of Cardiology (ESC). *Eur. Heart J.* **42**, 17–96 (2021). | ESC Guidelines |
| 59 | Gulati, M. *et al.* 2021 AHA/ACC/ASE/CHEST/SAEM/SCCT/SCMR Guideline for the evaluation and diagnosis of chest pain: a report of the American College of Cardiology/American Heart Association Joint Committee on Clinical Practice Guidelines. *J. Am. Coll. Cardiol.* **78**, e187–e285 (2021). | ACC Guidelines |
| 60 | Lawton, J.S. *et al.* 2021 ACC/AHA/SCAI Guideline for coronary artery revascularization: a report of the American College of Cardiology/American Heart Association Joint Committee on Clinical Practice Guidelines. *J. Am. Coll. Cardiol.* **79**, e21–e129 (2022). | ACC Guidelines |
| 61 | Maddox, T.M. *et al.* 2021 Update to the 2017 ACC Expert Consensus Decision Pathway for optimization of heart failure treatment: answers to 10 pivotal issues about heart failure with reduced ejection fraction: a report of the American College of Cardiology Solution Set Oversight Committee. *J. Am. Coll. Cardiol.* **77**, 772–810 (2021). | ACC expert consensus |
| 62 | Virani, S.S. *et al.* 2021 ACC Expert Consensus Decision Pathway on the management of ASCVD risk reduction in patients with persistent hypertriglyceridemia: a report of the American College of Cardiology Solution Set Oversight Committee. *J. Am. Coll. Cardiol.* **78**, 960–993 (2021). | ACC expert consensus |
| 63 | Rao, S.V. *et al.* 2021 ACC Expert Consensus Decision Pathway on same-day discharge after percutaneous coronary intervention: a report of the American College of Cardiology Solution Set Oversight Committee. *J. Am. Coll. Cardiol.* **77**, 811–825 (2021). | ACC expert consensus |
| 64 | Visseren, F.L.J. *et al.* 2021 ESC Guidelines on cardiovascular disease prevention in clinical practice: developed by the Task Force of the European Society of Cardiology (ESC) and 12 medical societies with the special contribution of the European Association of Preventive Cardiology (EAPC). *Eur. Heart J.* 42, 3227–3337 (2021). | ESC Guidelines |
| 65 | McDonagh, T.A. *et al.* 2021 ESC Guidelines for the diagnosis and treatment of acute and chronic heart failure: developed by the Task Force of the European Society of Cardiology (ESC) with the special contribution of the Heart Failure Association (HFA) of the ESC. *Eur. Heart J.* **42**, 3599–3726 (2021). | ESC Guidelines |
| 66 | Vahanian, A. *et al.* 2021 ESC/EACTS Guidelines for the management of valvular heart disease: developed by the Task Force of the European Society of Cardiology (ESC) and the European Association for Cardio-Thoracic Surgery (EACTS). *Eur. Heart J.* **43**, 561–632 (2022). | ESC Guidelines |
| 67 | Glikson, M. *et al.* 2021 ESC Guidelines on cardiac pacing and cardiac resynchronization therapy: developed by the Task Force of the European Society of Cardiology (ESC) with the special contribution of the European Heart Rhythm Association (EHRA). *Eur. Heart J.* **42**, 3427–3520 (2021). | ESC Guidelines |
| 68 | Isselbacher, E.M. *et al.* 2022 ACC/AHA Guideline for the Diagnosis and Management of Aortic Disease: a report of the American Heart Association/American College of Cardiology Joint Committee on Clinical Practice Guidelines. *J. Am. Coll. Cardiol.* **80**, 2001–2157 (2022). | ACC Guidelines |
| 69 | Birtcher, K.K. *et al.* 2022 ACC Expert Consensus Decision Pathway for Integrating Atherosclerotic Cardiovascular Disease and Multimorbidity Treatment: a framework for pragmatic, patient-centered care: a report of the American College of Cardiology Solution Set Oversight Committee. *J. Am. Coll. Cardiol.* **81**, 245–263 (2023). | ACC expert consensus |
| 70 | Cury, R.C. *et al.* CAD-RADS™ 2.0 – 2022 Coronary Artery Disease–Reporting and Data System: an expert consensus document of the Society of Cardiovascular Computed Tomography (SCCT), the American College of Cardiology (ACC), the American College of Radiology (ACR), and the North America Society of Cardiovascular Imaging (NASCI). *JACC Cardiovasc. Imaging* **15**, 1913–1932 (2022). | ACC expert consensus |
| 71 | Gluckman, T.J. *et al.* 2022 ACC Expert Consensus Decision Pathway on Cardiovascular Sequelae of COVID-19 in Adults: myocarditis and other myocardial involvement, post-acute sequelae of SARS-CoV-2 infection, and return to play: a report of the American College of Cardiology Solution Set Oversight Committee. *J. Am. Coll. Cardiol.* **79**, 1717–1756 (2022). | ACC expert consensus |
| 72 | Kontos, M.C. *et al.* 2022 ACC Expert Consensus Decision Pathway on the Evaluation and Disposition of Acute Chest Pain in the Emergency Department: a report of the American College of Cardiology Solution Set Oversight Committee. *J. Am. Coll. Cardiol.* **80**, 1925–1960 (2022). | ACC expert consensus |
| 73 | Lloyd-Jones, D.M. *et al.* 2022 ACC Expert Consensus Decision Pathway on the Role of Nonstatin Therapies for LDL-Cholesterol Lowering in the Management of Atherosclerotic Cardiovascular Disease Risk: a report of the American College of Cardiology Solution Set Oversight Committee. *J. Am. Coll. Cardiol.* **80**, 1366–1418 (2022). | ACC expert consensus |
| 74 | Heidenreich, P.A. *et al.* 2022 AHA/ACC/HFSA Guideline for the Management of Heart Failure: a report of the American College of Cardiology/American Heart Association Joint Committee on Clinical Practice Guidelines. *J. Am. Coll. Cardiol.* **79**, 1757–1850 (2022). | ACC Guidelines |
| 75 | Lyon, A.R. *et al.* 2022 ESC Guidelines on cardio-oncology: developed in collaboration with the European Hematology Association (EHA), the European Society for Therapeutic Radiology and Oncology (ESTRO), and the International Cardio-Oncology Society (IC-OS). *Eur. Heart J.* **43**, 4229–4361 (2022). | ESC Guidelines |
| 76 | Gorog, D.A. *et al.* Assessment and mitigation of bleeding risk in atrial fibrillation and venous thromboembolism: a position paper from the ESC Working Group on Thrombosis, in collaboration with the European Heart Rhythm Association, the Association for Acute CardioVascular Care, and the Asia-Pacific Heart Rhythm Society. *Europace* **24**, 1844–1871 (2022). | ESC consensus document |
| 77 | Wilde, A.A.M. *et al.* EHRA/HRS/APHRS/LAHRS expert consensus statement on the state of genetic testing for cardiac diseases. *Europace* **24**, 1307–1367 (2022). | ESC consensus document |
| 78 | Krychtiuk, K.A. *et al.* Acute LDL-C reduction post-ACS: strike early and strike strong—from evidence to clinical practice: a clinical consensus statement of the Association for Acute CardioVascular Care (ACVC), in collaboration with the European Association of Preventive Cardiology (EAPC) and the European Society of Cardiology Working Group on Cardiovascular Pharmacotherapy. *Eur. Heart J. Acute Cardiovasc. Care* **11**, 939–949 (2022). | ESC consensus document |
| 79 | Sade, L.E. *et al.* How to assess severe tricuspid regurgitation by echocardiography? *Eur. Heart J. Cardiovasc. Imaging* **23**, 1273–1276 (2022). | ESC consensus document |
| 80 | Pontone, G. *et al.* Clinical applications of cardiac computed tomography: a consensus paper of the European Association of Cardiovascular Imaging—part II. *Eur. Heart J. Cardiovasc. Imaging* **23**, e136–e161 (2022). | ESC consensus document |
| 81 | Halvorsen, S. *et al.* 2022 ESC Guidelines on cardiovascular assessment and management of patients undergoing non-cardiac surgery. *Eur. Heart J.* 43, 3826–3924 (2022). | ESC Guidelines |
| 82 | Zeppenfeld, K. *et al.* 2022 ESC Guidelines for the management of patients with ventricular arrhythmias and the prevention of sudden cardiac death. *Eur. Heart J.* **43**, 3997–4126 (2022). | ESC Guidelines |
| 83 | Humbert, M. *et al.* 2022 ESC/ERS Guidelines for the diagnosis and treatment of pulmonary hypertension. *Eur. Heart J.* **43**, 3618–3731 (2022). | ESC Guidelines |
| 84 | Joglar, J.A. *et al.* 2023 ACC/AHA/ACCP/HRS guideline for the diagnosis and management of atrial fibrillation: a report of the American College of Cardiology/American Heart Association Joint Committee on Clinical Practice Guidelines. *J. Am. Coll. Cardiol.* **83**, 85–227 (2024). | ACC Guidelines |
| 85 | Virani, S.S. *et al.* 2023 AHA/ACC/ACCP/ASPC/NLA/PCNA guideline for the management of patients with chronic coronary disease: a report of the American Heart Association/American College of Cardiology Joint Committee on Clinical Practice Guidelines. *J. Am. Coll. Cardiol.* **82**, 837–980 (2023). | ACC Guidelines |
| 86 | Kittleson, M.M. *et al.* 2023 ACC expert consensus decision pathway on comprehensive multidisciplinary care for the patient with cardiac amyloidosis: a report of the American College of Cardiology Solution Set Oversight Committee. *J. Am. Coll. Cardiol.* **81**, 1059–1081 (2023). | ACC expert consensus |
| 87 | Kittleson, M.M. *et al.* 2023 ACC expert consensus decision pathway on management of heart failure with preserved ejection fraction: a report of the American College of Cardiology Solution Set Oversight Committee. *J. Am. Coll. Cardiol.* **81**, 1725–1763 (2023). | ACC expert consensus |
| 88 | Byrne, R.A. *et al.* 2023 ESC Guidelines for the management of acute coronary syndromes. *Eur. Heart J.* **44**, 3720–3826 (2023). | ESC Guidelines |
| 89 | Marx, N. *et al.* 2023 ESC Guidelines for the management of cardiovascular disease in patients with diabetes. *Eur. Heart J.* **44**, 4043–4140 (2023). | ESC Guidelines |
| 90 | Arbelo, E. *et al.* 2023 ESC Guidelines for the management of cardiomyopathies. *Eur. Heart J*. **44**, 3503–3626 (2023). | ESC Guidelines |
| 91 | Niebauer, J. *et al.* Preinterventional frailty assessment in patients scheduled for cardiac surgery or transcatheter aortic valve implantation: a consensus statement of the European Association for Cardio-Thoracic Surgery (EACTS) and the European Association of Preventive Cardiology (EAPC) of the European Society of Cardiology (ESC). *Eur. J. Prev. Cardiol.* **31**, 146–181 (2024). | ESC consensus document |
| 92 | Valgimigli, M. *et al.* Antithrombotic treatment strategies in patients with established coronary atherosclerotic disease. *Eur. Heart J. Cardiovasc. Pharmacother.* **9**, 462–496 (2023). | ESC consensus document |
| 93 | Savelieva, I. *et al.* EHRA expert consensus document on the management of arrhythmias in frailty syndrome: endorsed by the Heart Rhythm Society (HRS), Asia Pacific Heart Rhythm Society (APHRS), Latin America Heart Rhythm Society (LAHRS), and Cardiac Arrhythmia Society of Southern Africa (CASSA). *Europace* **25**, 1249–1276 (2023). | ESC consensus document |
| 94 | Tarantini, G. *et al.* Management of coronary artery disease in patients undergoing transcatheter aortic valve implantation: a clinical consensus statement from the European Association of Percutaneous Cardiovascular Interventions in collaboration with the ESC Working Group on Cardiovascular Surgery. *EuroIntervention* **19**, 37–52 (2023). | ESC consensus document |
| 95 | Brida, M. *et al.* Acquired cardiovascular disease in adults with congenital heart disease: a call to action for timely preventive measures—a clinical consensus statement of the European Society of Cardiology Working Group on Adult Congenital Heart Disease in collaboration with the European Association of Preventive Cardiology and the European Association of Percutaneous Cardiovascular Interventions. *Eur. Heart J.* **44**, 4533–4548 (2023). | ESC consensus document |
| 96 | Musialek, P. *et al.* Stroke risk management in carotid atherosclerotic disease: a clinical consensus statement of the ESC Council on Stroke and the ESC Working Group on Aorta and Peripheral Vascular Diseases. *Cardiovasc. Res.* (2023). | ESC consensus document |
| 97 | Burri, H. *et al.* EHRA clinical consensus statement on conduction system pacing implantation: endorsed by the Asia Pacific Heart Rhythm Society (APHRS), Canadian Heart Rhythm Society (CHRS), and Latin American Heart Rhythm Society (LAHRS). *Europace* **25**, 1208–1236 (2023). | ESC consensus document |
| 98 | Jorge-Perez, P. *et al.* Management of comatose survivors of out-of-hospital cardiac arrest in Europe: current treatment practice and adherence to guidelines. *Eur. Heart J. Acute Cardiovasc. Care* **12**, 96–105 (2023). | ESC consensus document |
| 99 | Andreotti, F. *et al.* Acute, periprocedural and long-term antithrombotic therapy in older adults: 2022 update by the ESC Working Group on Thrombosis. *Eur. Heart J.* **44**, 262–279 (2023). | ESC consensus document |
| 100 | Barbato, E*. et al.* Renal denervation in the management of hypertension in adults: a clinical consensus statement of the ESC Council on Hypertension and the European Association of Percutaneous Cardiovascular Interventions (EAPCI). *Eur. Heart J.* **44**, 1313–1330 (2023). | ESC consensus document |
| 101 | Evangelista, A. *et al.* Multimodality imaging in thoracic aortic diseases: a clinical consensus statement from the European Association of Cardiovascular Imaging and the European Society of Cardiology Working Group on Aorta and Peripheral Vascular Diseases. *Eur. Heart J. Cardiovasc. Imaging* **24**, e65–e85 (2023). | ESC consensus document |
| 102 | Lang, I.M. *et al.* Balloon pulmonary angioplasty for chronic thromboembolic pulmonary hypertension: a clinical consensus statement of the ESC Working Group on Pulmonary Circulation and Right Ventricular Function. *Eur. Heart J.* **44**, 2659–2671 (2023). | ESC consensus document |
| 103 | Delgado, V. *et al.* 2023 ESC Guidelines for the management of endocarditis. *Eur. Heart J.* **44**, 3948–4042 (2023). | ESC Guidelines |
| 104 | McDonagh, T.A. *et al.* 2023 Focused Update of the 2021 ESC Guidelines for the diagnosis and treatment of acute and chronic heart failure. *Eur. Heart J.* **44**, 3627–3639 (2023). | ESC Guidelines |
| 105 | Kumbhani, D.J. *et al.* 2020 ACC expert consensus decision pathway for anticoagulant and antiplatelet therapy in patients with atrial fibrillation or venous thromboembolism undergoing percutaneous coronary intervention or with atherosclerotic cardiovascular disease: a report of the American College of Cardiology Solution Set Oversight Committee. *J. Am. Coll. Cardiol.* **77**, 629–658 (2021). | ACC expert consensus |
| 106 | Maddox, T.M. *et al.* 2024 ACC expert consensus decision pathway for treatment of heart failure with reduced ejection fraction: a report of the American College of Cardiology Solution Set Oversight Committee. *J. Am. Coll. Cardiol.* **83**, 1475–1501 (2024). | ACC expert consensus |
| 107 | Galassi, A.R. *et al.* Evaluation and management of patients with coronary chronic total occlusions considered for revascularisation: a clinical consensus statement of the European Association of Percutaneous Cardiovascular Interventions (EAPCI) of the ESC, the European Association of Cardiovascular Imaging (EACVI) of the ESC, and the ESC Working Group on Cardiovascular Surgery. *EuroIntervention* **20**, e174–e184 (2024). | ESC consensus document |
| 108 | Mazzolai, L. *et al.* Exercise therapy for chronic symptomatic peripheral artery disease: a clinical consensus document of the European Society of Cardiology Working Group on Aorta and Peripheral Vascular Diseases in collaboration with the European Society of Vascular Medicine and the European Society for Vascular Surgery. *Eur. Heart J.* **45**, 1–19 (2024). | ESC consensus document |
| 109 | Ali, N. *et al.* European Society of Cardiology quality indicators for the care and outcomes of adults undergoing transcatheter aortic valve implantation. *Eur. Heart J. Qual. Care Clin. Outcomes* **0**, 1–14 (2024). | ESC consensus document |
| 110 | *Cardiovascular Intervention: A Companion to Braunwald’s Heart Disease*  (Deepak L. Bhatt, 2015, Elsevier) | Textbook |
| 111 | *Clinical Lipidology: A Companion to Braunwald’s Heart Disease*, 2nd ed.  (Christie M. Ballantyne, 2015, Elsevier) | Textbook |
| 112 | *Chronic Coronary Artery Disease: A Companion to Braunwald’s Heart Disease*  (James A. de Lemos and Torbjørn Omland, 2018, Elsevier) | Textbook |
| 113 | *Arrhythmology and Electrophysiology: A Companion to Braunwald’s Heart Disease*, 3rd ed.  (Ziad F. Issa, John M. Miller, and Douglas P. Zipes, 2018, Elsevier) | Textbook |
| 114 | *Hypertension: A Companion to Braunwald’s Heart Disease*, 3rd ed.  (George L. Bakris and Matthew J. Sorrentino, 2018, Elsevier) | Textbook |
| 115 | *Myocardial Infarction: A Companion to Braunwald’s Heart Disease*  (David A. Morrow, 2018, Elsevier) | Textbook |
| 116 | *Essential Echocardiography: A Companion to Braunwald’s Heart Disease*  (Scott D. Solomon, Justina C. Wu, and Linda D. Gillam, 2018, Elsevier) | Textbook |
| 117 | *Heart Failure: A Companion to Braunwald’s Heart Disease*, 4th ed.  (G. Michael Felker and Douglas L. Mann, 2019, Elsevier) | Textbook |
| 118 | *Mechanical Circulatory Support: A Companion to Braunwald’s Heart Disease*, 2nd ed.  (James K. Kirklin and Joseph G. Rogers, 2019, Elsevier) | Textbook |
| 119 | *Cardiovascular Magnetic Resonance: A Companion to Braunwald’s Heart Disease*, 3rd ed.  (Warren J. Manning and Dudley J. Pennell, 2020, Elsevier) | Textbook |
| 120 | *Opie’s Cardiovascular Drugs: A Companion to Braunwald’s Heart Disease*, 9th ed.  (2020, Elsevier) | Textbook |
| 121 | *Valvular Heart Disease: A Companion to Braunwald’s Heart Disease*, 5th ed.  (Catherine M. Otto and Robert O. Bonow, 2020, Elsevier) | Textbook |
| 122 | *Vascular Medicine: A Companion to Braunwald’s Heart Disease*, 3rd ed.  (Mark A. Creager, Joshua A. Beckman, and Joseph Loscalzo, 2020, Elsevier) | Textbook |
| 123 | *Nuclear Cardiology and Multimodal Cardiovascular Imaging: A Companion to Braunwald’s Heart Disease*  (Marcelo F. Di Carli, 2020, Elsevier) | Textbook |

**S5 Table.** List of societal guidelines/consensus documents and cardiology textbooks in chronological order, which were included in the Knowledge Base as part of the Retrieval-Augmented Generation process.
